# How Do Medical Students Respond to Managing Co-Occurring Mental and Physical Health Problems in Simulated Settings? Insights for Medical Educators

**DOI:** 10.1192/bjo.2026.11283

**Published:** 2026-06-30

**Authors:** Thomas Rourke, Jamie Coleman, Erin Turner, Jo Protheroe, Magdy Abdulla

**Affiliations:** 1 Black Country Healthcare NHS Foundation Trust, Wolverhampton, United Kingdom; 2 Aston University, Birmingham, United Kingdom; 3 Birmingham University, Birmingham, United Kingdom; 4 Birmingham and Solihull Mental Health Trust, Birmingham, United Kingdom; 5 Keele University, Keele, United Kingdom

## Abstract

**Aims:**

Qualified doctors are required to manage complexity and uncertainty. This can arise with the co-occurrence of mental and physical health conditions in psychiatric settings. In response, this exploratory study was conducted to generate insights into how medical students respond to co-occurring mental and physical health problems in simulated psychiatric settings. It is hoped this can provide insights into how medical educators can better prepare the next generation of doctors for recognising and managing the complexity and uncertainty of encountering co-occurring mental and physical health problems in their clinical practice and increase awareness of the risk of diagnostic overshadowing, especially in the psychiatric patient population.

This study also seeks, through grounded thematic analysis, to gather and analyse the nuanced views of medical students at two diverse institutions on a simulation intervention; medical students’ nuanced views have historically been overlooked in medical educational interventions and are useful for medical educators to guide their practice.

**Methods:**

Fourth-year Birmingham and Aston medical students undertake a psychiatric simulation session. This high-fidelity session, which students undertake in pairs with the others observing, is on a simulated inpatient psychiatric ward with an actor as the simulated patient and facilitators as a psychiatric nurse and as senior support via telephone. One of the cases contained co-occurring mental and physical health problems. The researcher conducted the debrief of this case using a supplementary topic guide. These debriefs were recorded, transcribed and anonymised. A grounded thematic analysis of the resultant transcripts was then conducted.

**Results:**

Twelve debriefs were conducted involving 110 students. Grounded thematic analysis of transcripts resulted in three overriding themes: Using previous simulation experience, Recognising and managing feelings, and View of Psychiatry.

**Conclusion:**

The findings of this thematic analysis inform medical educators on how students respond and explain their responses to managing co-occurring physical and mental health problems in simulated psychiatric settings. The resultant discussion of these themes explores potential explanations for these responses, providing insights into how medical educators, especially within psychiatry, could better ensure that graduating medical students can manage complexity and uncertainty on qualification and increase awareness of the risk of diagnostic overshadowing, especially in the psychiatric patient population.

